# [(1R*,3S*,4S*)-3-(2-Hy­droxy­benzo­yl)-1,2,3,4-tetra­hydro-1,4-ep­oxy­naphthalen-1-yl]methyl 4-nitro­benzoate

**DOI:** 10.1107/S2414314620002655

**Published:** 2020-02-28

**Authors:** Alan J. Lough, Angel Ho, William Tam

**Affiliations:** a Department of Chemistry, University of Toronto, Toronto, Ontario, M5S 3H6, Canada; b Department of Chemistry, University of Guelph, Guelph, Ontario, N1G 2W1, Canada; University of Aberdeen, Scotland

**Keywords:** crystal structure, regioselectivity, weak hydrogen bonding

## Abstract

The relative stereo- and regiochemistry of the racemic title compound, C_25_H_19_NO_7_, were established from the crystal structure. The fused benzene ring forms dihedral angles of 77.3 (1) and 60.3 (1)° with the hy­droxy-substituted benzene ring and the nitro-substituted benzene ring, respectively. The dihedral angle between the hy­droxy-substituted benzene ring and the nitro-substituted benzene ring is 76.4 (1)°. An intra­molecular O—H⋯O hydrogen bond closes an *S*(6) ring. In the crystal, weak C—H⋯O hydrogen bonds connect the mol­ecules, forming layers parallel to (100). Within these layers, there are weak π–π stacking inter­actions with a ring centroid–ring centroid distance of 3.555 (1) Å.

## Abstract 2

The relative stereo- and regiochemistry of the title compound, C_25_H_19_O_7_N, were determined by the X-ray analysis.

## Graphical abstract

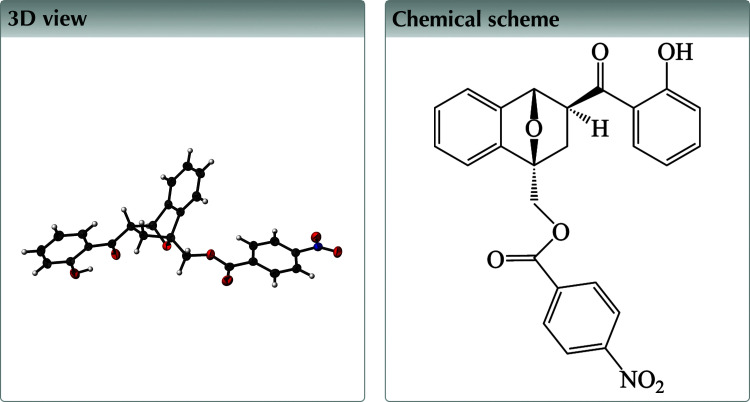

## Structure description

**Figure 1:**
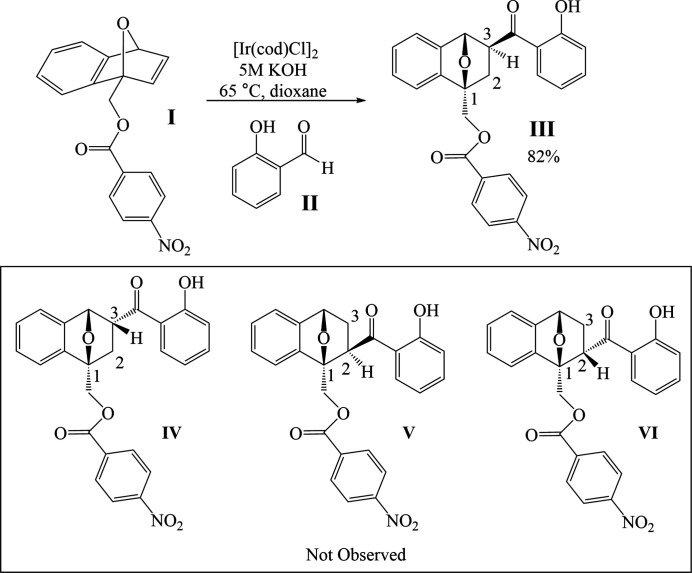
The reaction scheme.

In past years, our research group (Ballantine *et al.*, 2009 ▸; Edmunds *et al.*, 2015 ▸; Hill & Tam, 2019 ▸; Edmunds *et al.*, 2016 ▸; Raheem *et al.*, 2014 ▸) has investigated the effects of various C_1_-substituted oxabenzonorbornadienes (OBD) on controlling the regioselectivity of ring-opening reactions. In 2015 ▸, Nagamoto and Nishimura reported the iridium-catalysed hydro­acyl­ation reaction of bicyclic alkenes with 2-hy­droxy­benzaldehyde and its derivatives. Based upon these findings, we set out to determine the effect of C_1_ substitution on controlling the regioselectivity in the iridium-catalysed hydro­acyl­ation reaction with salicyl­aldehyde **II** (see Fig. 1 ▸) on unsymmetrical oxabenzonorbornadienes. Reaction of C_1_-substituted OBD (**I**) with salicyl­aldehyde **II** in the presence of [Ir(COD)Cl]_2_ (COD = 1,5-cyclo­octa­iene), and 5 *M* KOH afforded exclusively the title C_3_ regioisomer (**III**) in a 82% yield. The relative stereo- and regiochemistry of the adduct system was determined by single-crystal X-ray analysis. There are two possible stereoisomers as the addition can occur on the *exo* or the *endo* face, and two possible regioisomers as the addition can occur at the C_2_ or C_3_ position. Of the four possible stereo- and regio-isomers, only the *exo*-C_3_ isomer was obtained. The title compound is racemic: in the arbitrarily chosen asymmetric unit, the stereogenic centres are as follows: C1 *R*; C3 *S*; C4 *S*.

**Figure 2:**
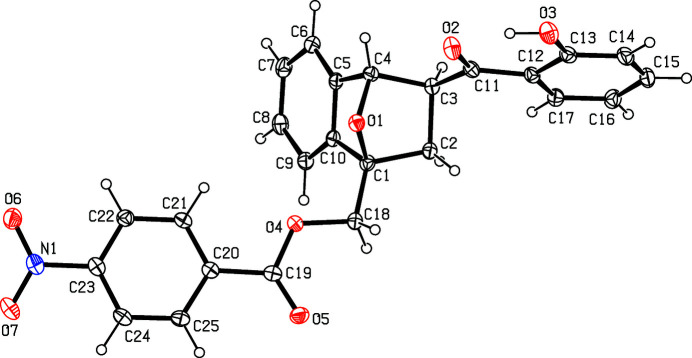
The mol­ecular structure of the title compound with displacement ellipsoids drawn the 30% probability level.

**Figure 3:**
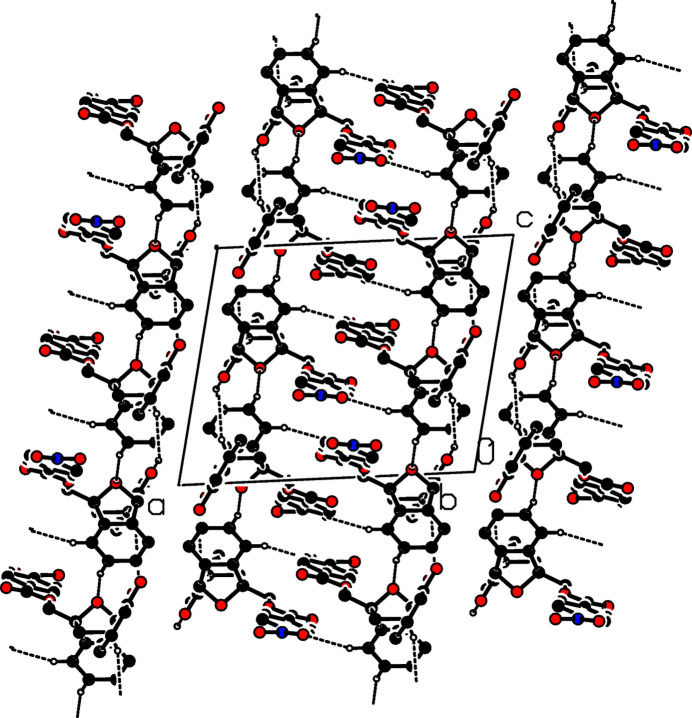
Part of the crystal structure with weak hydrogen bonds shown as dashed lines. Only H atoms involved in hydrogen bonds are shown.

The mol­ecular structure of the title compound is shown in Fig. 2 ▸. The fused benzene ring (C5–C10) forms dihedral angles of 77.3 (1) and 60.3 (1)° with the hy­droxy-substituted benzene ring (C12–C17) and the nitro-substituted benzene ring (C20–C25), respectively. The dihedral angle between the hy­droxy-substituted benzene ring and the nitro-substituted benzene ring is 76.4 (1)°. An intra­molecular O—H⋯O hydrogen bond is observed. In the crystal, weak C—H⋯O hydrogen bonds (Table 1 ▸) connect the mol­ecules, forming layers lying parallel to (100) (Fig. 3 ▸). Within these layers, there are weak π–π stacking inter­actions with a ring centroid–ring centroid distance of 3.555 (1) Å for *Cg*⋯*Cg*(1 − *x*, −*y*, 1 − *z*) where *Cg* is the centroid of the C20–C25 ring.

## Synthesis and crystallization

To a dried screw-cap vial, was added [Ir(COD)Cl]_2_ (10 mg, 5 mol%), C_1_-substituted oxabenzonorbornadiene (**I**) (Fig. 1 ▸) (0.3 mmol, 1.2 equiv.), salicyl­aldehyde **II** (27 µl, 1 equiv.) and 5*M* KOH (0.03 mmol, 10 mol%) dissolved in 2 ml of 1,4-dioxane. The reaction was left to stir at 338 K for 20 h, the resultant mixture was loaded directly onto a column and the crude reaction mixture was purified by flash chromatography (EtOAc:hexa­nes 25:75) to obtain the adduct product **III** (101 mg, 0.23 mmol, 82%) as a yellow solid. The product was then subsequently recrystallized from solution in pure hexa­nes by slow evaporation of the solvent to give product **III** as colourless crystals.

## Refinement

Crystal data, data collection and structure refinement details are summarized in Table 2 ▸.

## Supplementary Material

Crystal structure: contains datablock(s) I. DOI: 10.1107/S2414314620002655/hb4338sup1.cif

Structure factors: contains datablock(s) I. DOI: 10.1107/S2414314620002655/hb4338Isup2.hkl

Supporting information file. DOI: 10.1107/S2414314620002655/hb4338Isup3.cml

CCDC reference: 1986297

Additional supporting information:  crystallographic information; 3D view; checkCIF report

## Figures and Tables

**Table 1:** Hydrogen-bond geometry (Å, °)

*D*—H⋯*A*	*D*—H	H⋯*A*	*D*⋯*A*	*D*—H⋯*A*
O3—H3*O*⋯O2	0.94 (2)	1.67 (2)	2.5321 (15)	151.7 (19)
C3—H3*A*⋯O3^i^	1.00	2.48	3.4510 (17)	163
C8—H8*A*⋯O1^ii^	0.95	2.38	3.2782 (17)	157
C9—H9*A*⋯O7^iii^	0.95	2.49	3.4057 (19)	161

Symmetry codes: (i) 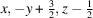 ; (ii) 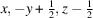 ; (iii) 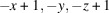 .

**Table 2:** Experimental details

Crystal data	
Chemical formula	C_25_H_19_NO_7_
*M*_r_	445.41
Crystal system, space group	Monoclinic, *P*2_1_/*c*
Temperature (K)	150
*a*, *b*, *c* (Å)	14.7455 (10), 11.9504 (8), 11.9649 (8)
β (°)	101.898 (2)
*V* (Å^3^)	2063.1 (2)
*Z*	4
Radiation type	Mo *K*α
μ (mm^−1^)	0.11
Crystal size (mm)	0.32 × 0.30 × 0.16

Data collection	
Diffractometer	Bruker Kappa APEX DUO PHOTON II
Absorption correction	Multi-scan (Krause *et al.*, 2015 ▸)
*T*_min_, *T*_max_	0.623, 0.746
No. of measured, independent and observed [*I* > 2σ(*I*)] reflections	40054, 4727, 3399
*R*_int_	0.059
(sin θ/λ)_max_ (Å^−1^)	0.650

Refinement	
*R*[*F* ^2^ > 2σ(*F* ^2^)], *wR*(*F* ^2^), *S*	0.036, 0.093, 1.01
No. of reflections	4727
No. of parameters	302
H-atom treatment	H atoms treated by a mixture of independent and constrained refinement
Δρ_max_, Δρ_min_ (e Å^−3^)	0.25, −0.21

Computer programs: *APEX3* and *SAINT* (Bruker, 2019 ▸), *SHELXT2014* (Sheldrick, 2015*a* ▸), *SHELXL2018* (Sheldrick, 2015*b* ▸), *PLATON* (Spek, 2020 ▸) and *publCIF* (Westrip, 2010 ▸).

## full crystallographic data

### Crystal data

**Table:**

C_25_H_19_NO_7_	*F*(000) = 928
*M**_r_* = 445.41	*D*_x_ = 1.434 Mg m^−^^3^
Monoclinic, *P*2_1_/*c*	Mo *K*α radiation, λ = 0.71073 Å
*a* = 14.7455 (10) Å	Cell parameters from 9213 reflections
*b* = 11.9504 (8) Å	θ = 2.4–27.2°
*c* = 11.9649 (8) Å	µ = 0.11 mm^−^^1^
β = 101.898 (2)°	*T* = 150 K
*V* = 2063.1 (2) Å^3^	Shard, colourless
*Z* = 4	0.32 × 0.30 × 0.16 mm

### Data collection

**Table:**

Bruker Kappa APEX DUO PHOTON II diffractometer	3399 reflections with *I* > 2σ(*I*)
Radiation source: sealed tube with Bruker Triumph monochromator	*R*_int_ = 0.059
φ and ω scans	θ_max_ = 27.5°, θ_min_ = 1.4°
Absorption correction: multi-scan (Krause *et al.*, 2015)	*h* = −19→19
*T*_min_ = 0.623, *T*_max_ = 0.746	*k* = −15→15
40054 measured reflections	*l* = −15→15
4727 independent reflections	

### Refinement

**Table:**

Refinement on *F*^2^	Primary atom site location: dual
Least-squares matrix: full	Hydrogen site location: mixed
*R*[*F*^2^ > 2σ(*F*^2^)] = 0.036	H atoms treated by a mixture of independent and constrained refinement
*wR*(*F*^2^) = 0.093	*w* = 1/[σ^2^(*F*_o_^2^) + (0.039*P*)^2^ + 0.5845*P*] where *P* = (*F*_o_^2^ + 2*F*_c_^2^)/3
*S* = 1.01	(Δ/σ)_max_ < 0.001
4727 reflections	Δρ_max_ = 0.25 e Å^−^^3^
302 parameters	Δρ_min_ = −0.21 e Å^−^^3^
0 restraints	

### Special details

**Table:**

Geometry. All esds (except the esd in the dihedral angle between two l.s. planes) are estimated using the full covariance matrix. The cell esds are taken into account individually in the estimation of esds in distances, angles and torsion angles; correlations between esds in cell parameters are only used when they are defined by crystal symmetry. An approximate (isotropic) treatment of cell esds is used for estimating esds involving l.s. planes.

### Fractional atomic coordinates and isotropic or equivalent isotropic displacement parameters (Å^2^)

**Table:**

	*x*	*y*	*z*	*U*_iso_*/*U*_eq_	
O1	0.21520 (6)	0.45410 (8)	0.52221 (8)	0.0254 (2)	
O2	0.09918 (8)	0.66077 (8)	0.55530 (9)	0.0357 (3)	
O3	0.08195 (8)	0.86474 (10)	0.60434 (9)	0.0376 (3)	
H3O	0.0802 (14)	0.7862 (18)	0.6067 (18)	0.069 (6)*	
O4	0.37771 (6)	0.32000 (8)	0.53811 (9)	0.0295 (2)	
O5	0.53204 (7)	0.32594 (9)	0.60060 (9)	0.0336 (2)	
O6	0.35631 (8)	−0.23766 (9)	0.63820 (10)	0.0430 (3)	
O7	0.50514 (8)	−0.25121 (9)	0.65612 (9)	0.0393 (3)	
N1	0.43308 (9)	−0.19667 (10)	0.64302 (10)	0.0317 (3)	
C1	0.27856 (9)	0.46052 (11)	0.44429 (11)	0.0239 (3)	
C2	0.26049 (9)	0.58196 (11)	0.39894 (12)	0.0247 (3)	
H2A	0.275088	0.590603	0.322264	0.030*	
H2B	0.296733	0.637046	0.451839	0.030*	
C3	0.15431 (9)	0.59376 (11)	0.39449 (11)	0.0238 (3)	
H3A	0.120016	0.605331	0.314073	0.029*	
C4	0.13143 (9)	0.47745 (11)	0.43864 (12)	0.0249 (3)	
H4A	0.072919	0.474098	0.468439	0.030*	
C5	0.13776 (9)	0.39344 (11)	0.34635 (12)	0.0249 (3)	
C6	0.07283 (10)	0.33873 (12)	0.26533 (13)	0.0302 (3)	
H6A	0.008319	0.348599	0.261361	0.036*	
C7	0.10549 (11)	0.26840 (12)	0.18949 (13)	0.0332 (3)	
H7A	0.062396	0.228731	0.133444	0.040*	
C8	0.19923 (11)	0.25522 (12)	0.19418 (12)	0.0319 (3)	
H8A	0.219548	0.206293	0.141757	0.038*	
C9	0.26456 (10)	0.31274 (11)	0.27481 (12)	0.0283 (3)	
H9A	0.329065	0.304773	0.277455	0.034*	
C10	0.23228 (9)	0.38140 (11)	0.35039 (11)	0.0235 (3)	
C11	0.13163 (9)	0.68588 (11)	0.47082 (11)	0.0254 (3)	
C12	0.14368 (9)	0.80400 (11)	0.44234 (12)	0.0250 (3)	
C13	0.11508 (10)	0.88844 (12)	0.50972 (12)	0.0283 (3)	
C14	0.11990 (10)	1.00024 (12)	0.47954 (14)	0.0349 (4)	
H14A	0.100414	1.056998	0.524970	0.042*	
C15	0.15290 (11)	1.02872 (12)	0.38389 (14)	0.0369 (4)	
H15A	0.155158	1.105273	0.363255	0.044*	
C16	0.18300 (10)	0.94724 (12)	0.31691 (13)	0.0340 (3)	
H16A	0.206590	0.967947	0.251714	0.041*	
C17	0.17825 (10)	0.83600 (12)	0.34613 (12)	0.0287 (3)	
H17A	0.198678	0.780174	0.300444	0.034*	
C18	0.37610 (10)	0.43650 (11)	0.50427 (12)	0.0279 (3)	
H18A	0.419456	0.449528	0.452497	0.034*	
H18B	0.394223	0.485260	0.572100	0.034*	
C19	0.46027 (9)	0.27418 (12)	0.57989 (11)	0.0253 (3)	
C20	0.45169 (9)	0.15105 (11)	0.59774 (11)	0.0247 (3)	
C21	0.36672 (9)	0.09770 (12)	0.56869 (12)	0.0274 (3)	
H21A	0.312763	0.139404	0.536667	0.033*	
C22	0.36020 (10)	−0.01641 (12)	0.58624 (12)	0.0287 (3)	
H22A	0.302095	−0.053641	0.568005	0.034*	
C23	0.44020 (10)	−0.07438 (12)	0.63079 (11)	0.0273 (3)	
C24	0.52603 (10)	−0.02386 (12)	0.65983 (12)	0.0293 (3)	
H24A	0.579934	−0.066435	0.689955	0.035*	
C25	0.53146 (10)	0.09052 (12)	0.64388 (12)	0.0284 (3)	
H25A	0.589463	0.127695	0.664342	0.034*	

### Atomic displacement parameters (Å^2^)

**Table:**

	*U*^11^	*U*^22^	*U*^33^	*U*^12^	*U*^13^	*U*^23^
O1	0.0287 (5)	0.0252 (5)	0.0235 (5)	0.0008 (4)	0.0085 (4)	0.0022 (4)
O2	0.0536 (7)	0.0288 (5)	0.0292 (5)	0.0063 (5)	0.0189 (5)	0.0028 (4)
O3	0.0493 (7)	0.0330 (6)	0.0332 (6)	0.0069 (5)	0.0145 (5)	−0.0052 (5)
O4	0.0247 (5)	0.0248 (5)	0.0380 (6)	0.0019 (4)	0.0040 (4)	0.0041 (4)
O5	0.0259 (5)	0.0370 (6)	0.0358 (6)	−0.0032 (5)	0.0019 (4)	0.0034 (5)
O6	0.0423 (7)	0.0330 (6)	0.0484 (7)	−0.0031 (5)	−0.0029 (5)	0.0024 (5)
O7	0.0485 (7)	0.0354 (6)	0.0344 (6)	0.0168 (5)	0.0097 (5)	0.0035 (5)
N1	0.0408 (8)	0.0302 (7)	0.0224 (6)	0.0058 (6)	0.0027 (5)	−0.0004 (5)
C1	0.0258 (7)	0.0230 (7)	0.0245 (7)	−0.0012 (5)	0.0085 (6)	0.0011 (5)
C2	0.0277 (7)	0.0212 (6)	0.0260 (7)	−0.0009 (5)	0.0074 (6)	0.0007 (5)
C3	0.0272 (7)	0.0219 (7)	0.0228 (7)	0.0008 (5)	0.0059 (5)	0.0002 (5)
C4	0.0236 (7)	0.0241 (7)	0.0278 (7)	0.0015 (5)	0.0072 (6)	0.0017 (6)
C5	0.0278 (7)	0.0193 (6)	0.0280 (7)	−0.0008 (5)	0.0069 (6)	0.0025 (5)
C6	0.0279 (7)	0.0254 (7)	0.0360 (8)	−0.0022 (6)	0.0036 (6)	0.0018 (6)
C7	0.0402 (9)	0.0258 (7)	0.0307 (8)	−0.0053 (6)	0.0007 (7)	−0.0019 (6)
C8	0.0441 (9)	0.0242 (7)	0.0284 (7)	0.0012 (6)	0.0098 (7)	−0.0032 (6)
C9	0.0311 (8)	0.0251 (7)	0.0305 (8)	0.0010 (6)	0.0105 (6)	0.0011 (6)
C10	0.0265 (7)	0.0202 (6)	0.0242 (7)	−0.0012 (5)	0.0058 (5)	0.0031 (5)
C11	0.0273 (7)	0.0263 (7)	0.0220 (7)	0.0039 (6)	0.0036 (6)	0.0020 (6)
C12	0.0250 (7)	0.0240 (7)	0.0239 (7)	0.0026 (5)	0.0003 (5)	0.0000 (5)
C13	0.0268 (7)	0.0280 (7)	0.0279 (7)	0.0035 (6)	0.0010 (6)	−0.0021 (6)
C14	0.0317 (8)	0.0259 (7)	0.0440 (9)	0.0040 (6)	0.0005 (7)	−0.0062 (7)
C15	0.0372 (9)	0.0223 (7)	0.0460 (9)	0.0010 (6)	−0.0032 (7)	0.0046 (7)
C16	0.0354 (8)	0.0305 (8)	0.0338 (8)	−0.0035 (6)	0.0015 (7)	0.0066 (6)
C17	0.0307 (7)	0.0267 (7)	0.0269 (7)	−0.0006 (6)	0.0018 (6)	0.0006 (6)
C18	0.0282 (7)	0.0227 (7)	0.0322 (8)	−0.0011 (6)	0.0044 (6)	0.0022 (6)
C19	0.0237 (7)	0.0326 (8)	0.0197 (7)	0.0025 (6)	0.0049 (5)	−0.0008 (6)
C20	0.0265 (7)	0.0281 (7)	0.0200 (6)	0.0027 (6)	0.0060 (5)	−0.0010 (5)
C21	0.0244 (7)	0.0293 (7)	0.0286 (7)	0.0057 (6)	0.0053 (6)	−0.0008 (6)
C22	0.0269 (7)	0.0287 (7)	0.0305 (7)	0.0004 (6)	0.0058 (6)	−0.0025 (6)
C23	0.0336 (8)	0.0277 (7)	0.0208 (7)	0.0061 (6)	0.0062 (6)	−0.0002 (5)
C24	0.0289 (8)	0.0335 (8)	0.0243 (7)	0.0090 (6)	0.0030 (6)	0.0004 (6)
C25	0.0248 (7)	0.0344 (8)	0.0251 (7)	0.0023 (6)	0.0035 (6)	−0.0018 (6)

### Geometric parameters (Å, º)

**Table:**

O1—C4	1.4471 (16)	C8—C9	1.396 (2)
O1—C1	1.4515 (15)	C8—H8A	0.9500
O2—C11	1.2410 (16)	C9—C10	1.3766 (19)
O3—C13	1.3523 (18)	C9—H9A	0.9500
O3—H3O	0.94 (2)	C11—C12	1.4715 (19)
O4—C19	1.3345 (16)	C12—C17	1.405 (2)
O4—C18	1.4487 (16)	C12—C13	1.4093 (19)
O5—C19	1.2066 (16)	C13—C14	1.390 (2)
O6—N1	1.2238 (16)	C14—C15	1.375 (2)
O7—N1	1.2287 (15)	C14—H14A	0.9500
N1—C23	1.4746 (19)	C15—C16	1.391 (2)
C1—C18	1.4968 (19)	C15—H15A	0.9500
C1—C10	1.5185 (18)	C16—C17	1.380 (2)
C1—C2	1.5531 (18)	C16—H16A	0.9500
C2—C3	1.5620 (19)	C17—H17A	0.9500
C2—H2A	0.9900	C18—H18A	0.9900
C2—H2B	0.9900	C18—H18B	0.9900
C3—C11	1.5112 (18)	C19—C20	1.4959 (19)
C3—C4	1.5486 (18)	C20—C21	1.3848 (19)
C3—H3A	1.0000	C20—C25	1.3942 (19)
C4—C5	1.5095 (19)	C21—C22	1.386 (2)
C4—H4A	1.0000	C21—H21A	0.9500
C5—C6	1.3788 (19)	C22—C23	1.3770 (19)
C5—C10	1.3920 (19)	C22—H22A	0.9500
C6—C7	1.393 (2)	C23—C24	1.380 (2)
C6—H6A	0.9500	C24—C25	1.385 (2)
C7—C8	1.381 (2)	C24—H24A	0.9500
C7—H7A	0.9500	C25—H25A	0.9500
			
C4—O1—C1	96.71 (9)	O2—C11—C12	120.38 (12)
C13—O3—H3O	104.6 (13)	O2—C11—C3	119.12 (12)
C19—O4—C18	117.40 (11)	C12—C11—C3	120.43 (12)
O6—N1—O7	124.11 (13)	C17—C12—C13	118.41 (13)
O6—N1—C23	118.42 (12)	C17—C12—C11	122.17 (12)
O7—N1—C23	117.46 (13)	C13—C12—C11	119.33 (13)
O1—C1—C18	111.35 (11)	O3—C13—C14	117.75 (13)
O1—C1—C10	101.05 (10)	O3—C13—C12	122.09 (13)
C18—C1—C10	118.44 (11)	C14—C13—C12	120.16 (14)
O1—C1—C2	100.78 (10)	C15—C14—C13	119.99 (14)
C18—C1—C2	115.17 (11)	C15—C14—H14A	120.0
C10—C1—C2	107.74 (11)	C13—C14—H14A	120.0
C1—C2—C3	101.24 (10)	C14—C15—C16	121.06 (14)
C1—C2—H2A	111.5	C14—C15—H15A	119.5
C3—C2—H2A	111.5	C16—C15—H15A	119.5
C1—C2—H2B	111.5	C17—C16—C15	119.31 (15)
C3—C2—H2B	111.5	C17—C16—H16A	120.3
H2A—C2—H2B	109.3	C15—C16—H16A	120.3
C11—C3—C4	110.90 (11)	C16—C17—C12	121.05 (14)
C11—C3—C2	112.98 (11)	C16—C17—H17A	119.5
C4—C3—C2	101.22 (10)	C12—C17—H17A	119.5
C11—C3—H3A	110.5	O4—C18—C1	106.08 (11)
C4—C3—H3A	110.5	O4—C18—H18A	110.5
C2—C3—H3A	110.5	C1—C18—H18A	110.5
O1—C4—C5	101.80 (10)	O4—C18—H18B	110.5
O1—C4—C3	101.18 (10)	C1—C18—H18B	110.5
C5—C4—C3	107.26 (11)	H18A—C18—H18B	108.7
O1—C4—H4A	115.0	O5—C19—O4	124.08 (13)
C5—C4—H4A	115.0	O5—C19—C20	124.85 (12)
C3—C4—H4A	115.0	O4—C19—C20	111.08 (11)
C6—C5—C10	121.37 (13)	C21—C20—C25	120.42 (13)
C6—C5—C4	133.71 (13)	C21—C20—C19	121.05 (12)
C10—C5—C4	104.82 (11)	C25—C20—C19	118.53 (12)
C5—C6—C7	117.43 (13)	C20—C21—C22	120.16 (13)
C5—C6—H6A	121.3	C20—C21—H21A	119.9
C7—C6—H6A	121.3	C22—C21—H21A	119.9
C8—C7—C6	121.32 (14)	C23—C22—C21	118.22 (13)
C8—C7—H7A	119.3	C23—C22—H22A	120.9
C6—C7—H7A	119.3	C21—C22—H22A	120.9
C7—C8—C9	120.95 (13)	C22—C23—C24	123.04 (14)
C7—C8—H8A	119.5	C22—C23—N1	117.66 (13)
C9—C8—H8A	119.5	C24—C23—N1	119.26 (12)
C10—C9—C8	117.70 (13)	C23—C24—C25	118.24 (13)
C10—C9—H9A	121.2	C23—C24—H24A	120.9
C8—C9—H9A	121.2	C25—C24—H24A	120.9
C9—C10—C5	121.20 (13)	C24—C25—C20	119.91 (13)
C9—C10—C1	133.92 (12)	C24—C25—H25A	120.0
C5—C10—C1	104.80 (11)	C20—C25—H25A	120.0
			
C4—O1—C1—C18	−178.46 (11)	O2—C11—C12—C17	−178.58 (13)
C4—O1—C1—C10	−51.79 (11)	C3—C11—C12—C17	−1.5 (2)
C4—O1—C1—C2	58.90 (11)	O2—C11—C12—C13	−2.1 (2)
O1—C1—C2—C3	−35.63 (12)	C3—C11—C12—C13	174.94 (12)
C18—C1—C2—C3	−155.56 (11)	C17—C12—C13—O3	−179.28 (12)
C10—C1—C2—C3	69.79 (12)	C11—C12—C13—O3	4.1 (2)
C1—C2—C3—C11	118.93 (12)	C17—C12—C13—C14	1.1 (2)
C1—C2—C3—C4	0.30 (12)	C11—C12—C13—C14	−175.51 (13)
C1—O1—C4—C5	51.61 (11)	O3—C13—C14—C15	−179.85 (13)
C1—O1—C4—C3	−58.89 (11)	C12—C13—C14—C15	−0.2 (2)
C11—C3—C4—O1	−84.83 (12)	C13—C14—C15—C16	−0.9 (2)
C2—C3—C4—O1	35.28 (12)	C14—C15—C16—C17	1.0 (2)
C11—C3—C4—C5	168.93 (11)	C15—C16—C17—C12	−0.1 (2)
C2—C3—C4—C5	−70.96 (12)	C13—C12—C17—C16	−0.9 (2)
O1—C4—C5—C6	151.71 (15)	C11—C12—C17—C16	175.55 (13)
C3—C4—C5—C6	−102.49 (17)	C19—O4—C18—C1	171.18 (11)
O1—C4—C5—C10	−32.04 (13)	O1—C1—C18—O4	66.60 (13)
C3—C4—C5—C10	73.76 (13)	C10—C1—C18—O4	−49.87 (15)
C10—C5—C6—C7	1.7 (2)	C2—C1—C18—O4	−179.47 (11)
C4—C5—C6—C7	177.47 (14)	C18—O4—C19—O5	6.95 (19)
C5—C6—C7—C8	−0.9 (2)	C18—O4—C19—C20	−173.23 (11)
C6—C7—C8—C9	−0.5 (2)	O5—C19—C20—C21	−178.22 (13)
C7—C8—C9—C10	1.0 (2)	O4—C19—C20—C21	1.96 (18)
C8—C9—C10—C5	−0.2 (2)	O5—C19—C20—C25	1.4 (2)
C8—C9—C10—C1	−176.44 (14)	O4—C19—C20—C25	−178.39 (11)
C6—C5—C10—C9	−1.2 (2)	C25—C20—C21—C22	0.6 (2)
C4—C5—C10—C9	−178.00 (12)	C19—C20—C21—C22	−179.80 (13)
C6—C5—C10—C1	176.00 (12)	C20—C21—C22—C23	−1.2 (2)
C4—C5—C10—C1	−0.82 (13)	C21—C22—C23—C24	0.8 (2)
O1—C1—C10—C9	−150.10 (15)	C21—C22—C23—N1	−176.67 (12)
C18—C1—C10—C9	−28.3 (2)	O6—N1—C23—C22	−16.19 (19)
C2—C1—C10—C9	104.67 (17)	O7—N1—C23—C22	162.95 (12)
O1—C1—C10—C5	33.25 (12)	O6—N1—C23—C24	166.29 (13)
C18—C1—C10—C5	155.09 (12)	O7—N1—C23—C24	−14.57 (18)
C2—C1—C10—C5	−71.98 (13)	C22—C23—C24—C25	0.4 (2)
C4—C3—C11—O2	−0.02 (17)	N1—C23—C24—C25	177.78 (12)
C2—C3—C11—O2	−112.86 (14)	C23—C24—C25—C20	−1.1 (2)
C4—C3—C11—C12	−177.14 (12)	C21—C20—C25—C24	0.6 (2)
C2—C3—C11—C12	70.02 (15)	C19—C20—C25—C24	−179.03 (12)

### Hydrogen-bond geometry (Å, º)

**Table:**

*D*—H···*A*	*D*—H	H···*A*	*D*···*A*	*D*—H···*A*
O3—H3*O*···O2	0.94 (2)	1.67 (2)	2.5321 (15)	151.7 (19)
C3—H3*A*···O3^i^	1.00	2.48	3.4510 (17)	163
C8—H8*A*···O1^ii^	0.95	2.38	3.2782 (17)	157
C9—H9*A*···O7^iii^	0.95	2.49	3.4057 (19)	161

Symmetry codes: (i) *x*, −*y*+3/2, *z*−1/2; (ii) *x*, −*y*+1/2, *z*−1/2; (iii) −*x*+1, −*y*, −*z*+1.
